# Pathology of the Osseous Structure of the Teeth

**Published:** 1881-03

**Authors:** A. J. Douds

**Affiliations:** Canton, Ohio


### PATHOLOGY OF THE OSSEOUS STRUCTURE OF THE TEETH.

BY A. J. DOUDS, CANTON, OHIO.

In the consideration of this subject, numerous pathological conditions present themselves to the mind, *all* requiring careful thought, and the field being a wide one we can only in this brief paper consider a single one of the pathological conditions of tooth structure, one to be met with in almost every day practice, and which in the effort to arrest, the dentist has experienced many mortifying failures, and which will be readily recognized when we mention the erosion of the enamel and decay of the teeth, during the period of gestation.

Who has not found his utmost efforts to save the teeth under this condition, result in inglorious failure, notwithstanding our boasted attainments, and how often has the distressed patient called in vain for relief, and our hands have hung down helplessly, and the humiliating confession has been extorted, that we have done our utmost and failed, and for this reason we are led to study more closely the causes of failure in the hope that we may be enabled to obtain better results in the future.

The local symptoms attending this condition may vary very greatly so far as the quantity of the salivary and mucous secretions are concerned, but usually present the ropy or stringy condition, but however the quantity may be affected by the greater or lesser excitability of the nervous system of the patient, the strong acid reaction is an invariable accompaniment of the disorder, with resultant extreme sensibility of the approximal surfaces and along the margin of the gums, followed by softening of the enamel and rapid decay of the dentine, often following the line of the tubuli causing unexpected exposure of the pulp.

These conditions are not, however, confined to the period referred to in all cases, but as a result of the exhausted nervous condition of the patient may continue during lactation and where this is the case the possibility of relief becomes much more uncertain and difficult, as the recuperative powers of the body are directed to the supply of milk.

The pathological conditions here enumerated present a very striking similarity to those presented in teeth of the young at the age of from 12 to 18 years, sex having but little to do in determining the liability of the teeth to this destructive agency.

Close observation extending over many years has brought this fact to notice, that the great preponderance of these cases are found in persons of a strong nervous temperament and its blendings.

And the question very naturally arises, has temperament any predisposing influence in bringing about this condition, and has it anything to do with the marked difference in the physical powers which strengthen or weaken the resisting capacity of the organism.

Predisposing causes of disease according to Wedl, consist of circumstances which influence functions and structure unfavorably yet without actual disease, and eminent writers on pathology of disease agree in enumerating among other predisposing causes, debilitating influences—hereditary constitution, and temperament.

In the nervous temperament there is great excitability and the preponderance of the emotions over the reason, and the will, and only those who are brought in contact with this class of patients can fully realize the varied mental conditions and their influence upon the general health.

The commonly accepted theory as to the cause of the rapid breaking down of the osseous structure of the teeth during this period, (viz. gestation) is that it is due to defective nutrition, in that the food lacking the elements of bone tissue, the osseous system new organism being built up makes demands which can only be supplied by the osseous system of the mother.

It is with the truth or falsity of this theory that we have to deal in this brief paper, and believing the facts to be at variance with the theory it must stand or fall according to its own merits.

And assuming that parturition is a perfectly normal and physiological act in harmony with the designs of procreation, how shall we be able to reconcile a few facts which we present.

Two women placed under similar surroundings, eating at the same table, partaking of the same food will present conditions diametrically opposite, the one robust in health, giving no evidence of undue irritability, the secretions of the mouth normal in quantity and quality, while the other exhibiting all the symptoms described.

Another fact is the want of evidence, that after the bones have lost the vascularity characteristic of them in youth and infantile periods, that there is any loss of osseous tissue through the medium of the absorbents to any appreciable extent, but on the contrary there is a constant increase in the density of the bones consequent upon the substitution of the inorganic for the organic constituents.

The pathological condition called rickets is not found in the adult, but in the young only, while from the great vascularity of the bony structure, the absorbents act readily and promptly, and the affection is readily relieved by proper diet and remedies.

This disease is found ordinarily only in the lowest class of society, and will not apply to the great mass of patients coming under the notice of the physician and dentist, though Carpenter in his physiology gives several instances where the affection was plainly due to menstruation during lactation, one case where only one of six children of a healthy mother presented this condition and in which during the nursing period the catamenial flow was constant.

That the erosion of the enamel is not due to interstitial absorption, is shown by the fact that the microscope has revealed no capillaries which could possibly transmit the fluids of the blood, and no nerves, therefore it is a purely inorganic body not subject to either nutritive or destructive assimilation.

Local lesions are in great part but the manifestation of systematic disturbances caused by deficient or excessive irritability, which is a vital property possessed by all living organized bodies capable of being acted upon and responding thereto.

If the conclusions drawn, then, be correct, we must seek another solution of the causes of the phenomena exhibited in the rapid destruction of the teeth in the condition under consideration.

As has been observed, the majority of cases in which these pathological conditions are found are in females where the nervous is the predominating type.

The researches of eminent physiologists have clearly demonstrated that nervous irritability, fright, anxiety, long continued mental strain, or depressed mental states, affect through the medium of the sympathetic nervous system such changes in the normal constituents of the secretions as to render them positively poisonous, as shown in the interesting cases cited by Dr. Carpenter in his Physiology, pages 739–40.

These facts have been so fully established that no further proof need be adduced than to refer to the effect of the changed condition of the milk in many of the cases under consideration, and which are readily recognized by its action upon the delicate apparatus of the infant, producing violent colicky pains and intestinal irritation and fever.

To nervous irritability, then, modified by temperament and habit, are we to look for a solution of the pathological condition of the teeth during that period of gestation, and it is believed that careful observation will furnish ample proof of the correctness of the theory.

Several striking instances illustrating this point might be given was there not danger of extending this paper to an undue length.

We need, then, only state that having correctly diagnosed the origin of the trouble, a rational mode of treatment will readily suggest itself.

Mild tonics, nervous sedatives, a generous, wholesome and nutritious diet, with fresh air and exercise, and as far as possible agreeable associations and surroundings, in fact everything calculated to induce a placid temper.

Not neglecting such local treatment as may be deemed necessary, avoiding long, exhausting operations, and where filling is necessary, gutta percha stoppings are frequently preferable, being durable and easily introduced.

This, together with a kind, sympathetic demeanor, will greatly alleviate the suffering, and in many cases prolong the usefulness of these organs so indispensable to health and beauty, and thus conferring substantial benefits we will earn the confidence and gratitude of our patients and realize that self-satisfaction which after all is the fee of all fees.—*Trans - Ohio State Dental Society.*

